# Thermography in Bike Fitting: A Literature Review

**DOI:** 10.3390/s25082356

**Published:** 2025-04-08

**Authors:** Warre Clarys, Oscar Vallet, Jan Verstockt, Hai Zhang, Simon Verspeek, Gunther Steenackers

**Affiliations:** 1 InViLab Research Group, University of Antwerp, Groenenborgerlaan 171, B-2020 Antwerp, Belgium; oscar.vallet@student.uantwerpen.be (O.V.); jan.verstockt@uantwerpen.be (J.V.); simon.verspeek@uantwerpen.be (S.V.); gunther.steenackers@uantwerpen.be (G.S.); 2 Computer Vision and Systems Laboratory (CVSL), Department of Electrical and Computer Engineering, Laval University, Quebec, QC G1V 0A6, Canada; hai.zhang.1@ulaval.ca

**Keywords:** infrared thermography, bike fitting, cycling biomechanics, thermal asymmetry, pressure distribution, injury prevention

## Abstract

Cycling comfort significantly impacts both enjoyment and performance, with discomfort potentially leading to injuries. Traditional bike-fitting methods, while effective for biomechanical adjustments, often overlook physiological responses such as pressure distribution and asymmetry. Infrared thermography (IRT), a non-invasive technique analyzing surface temperature variations, offers the potential to enhance bike fitting by identifying pressure points, asymmetries, and discomfort zones. This review evaluates the application of IRT in bike fitting, highlighting its ability to provide objective, real-time insights into cyclist comfort and injury prevention. However, limitations such as variability in thermographic protocols and the lack of standardized methodologies necessitate further research. By addressing these gaps, IRT could transform bike fitting into a more precise, personalized, and effective practice for cyclists across all levels.

## 1. Introduction

Cycling comfort is a fundamental factor influencing both the enjoyment and performance of cyclists. Physical discomfort—manifesting as numbness, pain, or improper saddle height—can significantly diminish the quality of the cycling experience [1]. For recreational cyclists, such discomforts may lead to injuries over time, potentially hindering their long-term engagement with the sport. Van der Walt et al. [2] reported that non-traumatic injuries occur in up to 88% of cyclists, resulting from a combination of factors including incorrect cycling posture, prolonged pressure, and suboptimal bike setup. According to Bini et al. [3], such posture-related issues can lead to the overloading of muscles and joints, causing common complaints such as lower back pain, neck discomfort, and knee problems. Knee injuries, in particular, are frequently severe, often necessitating medical intervention [2]. Addressing these issues through effective bike fitting is, therefore, essential to enhance rider comfort, optimize performance, and prevent injuries.

Bike fitting aims to align the rider’s position with the bike to enhance comfort, performance, and efficiency. While traditional fitting methods rely on manual measurements and visual assessments, recent advances have introduced technologies such as 3D scanning systems and inertial measurement unit (IMU) sensors for more precise and personalized outcomes. However, the lack of standardization across fitting studios often results in inconsistent recommendations [4]. Even well-calibrated adjustments may fall short by overlooking dynamic physiological factors like pressure points or bodily asymmetries, potentially reducing the effectiveness of the fit [4]. As Bini [5] noted, further research is needed—particularly on asymmetries and their influence on performance and injury prevention.

To address these gaps, various technologies are currently employed in bike fitting, each offering unique benefits. Motion capture systems, both marker-based and markerless, enable detailed kinematic analysis and are widely used in professional settings [6]. Pressure mapping tools provide insights into force distribution at key contact points, such as the saddle, handlebars, and pedals [7]. Complementary tools like 3D scanners and IMUs contribute to building individualized biomechanical models [8]. Although these methods yield valuable mechanical data, they often require specialized setups and may overlook physiological responses—such as tissue stress, heat buildup, and inflammation—that are critical for assessing comfort and injury risk [9].

**Figure 1:**
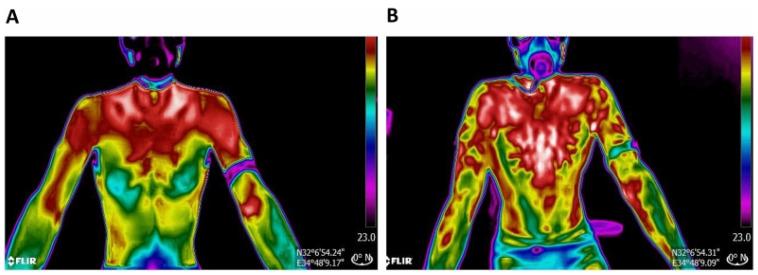
Thermal images of a cyclist’s torso at (**A**) rest and (**B**) after a strenuous cycling exercise. Warmer areas (in red) form a “tree-shaped” pattern on the chest and arms under intense effort, reflecting increased skin temperature from blood flow and muscle activity. IRT can reveal these thermal patterns, which may indicate high stress or pressure zones relevant to bike fit adjustments [24].

In this context, infrared thermography (IRT) offers a promising complementary approach. As a non-contact imaging technique, IRT measures surface temperature variations based on infrared radiation emitted by the body, as described by Planck’s law. These temperature variations can reflect underlying physiological processes influenced by pressure, muscle activation, or stress (Figure 1). IRT’s effectiveness stems from the principle that skin temperature reflects underlying physiological activity. During physical exertion, active muscles produce metabolic heat, resulting in increased skin temperature over those regions [10]. Similarly, pressure and friction at contact points—such as the saddle, pedals, and handlebars—can lead to localized thermal increases [11]. These heat patterns can be visualized through IRT, offering insight into pressure distribution, asymmetrical loading, and potential discomfort zones. Moreover, asymmetric muscle loading or improper posture can lead to thermal asymmetries exceeding 0.5 °C [12], indicating imbalanced effort or stress. By capturing these variations, IRT enables indirect assessment of metabolic effort, pressure distribution, and asymmetrical loading. IRT has already proven useful in fields such as medical diagnostics, engineering, and sports, including applications in running, strength training [13], rowing [14], soccer [15], and cycling [10,16,17,18]. Its ability to non-invasively detect asymmetries and pressure points makes it particularly relevant to cycling comfort and injury prevention [12,17,19,20,21,22,23,24].

When applied to bike fitting, IRT has the potential to enhance objectivity by identifying physiological differences, such as those between male and female cyclists—a critical area where research on women remains comparatively limited [25,26,27]. Most bicycles and bike-fitting methodologies have historically been designed based on male anatomy and biomechanics, often overlooking key differences in pelvic structure, muscle activation patterns, and pressure distribution [25]. Addressing these differences could lead to improved comfort and more tailored outcomes, particularly for female cyclists, who may experience higher discomfort and inefficiencies due to improper fit.

However, only one study has directly examined the integration of IRT into bike-fitting practices [10], despite 318 bike-fitting-related articles being published since 2015 on the Web of Science. Therefore, the purpose of this systematic review is to evaluate how IRT can be applied in bike fitting and to determine its effectiveness in identifying cycling discomfort and injury risk factors. By clarifying the current evidence base, this review aims to guide future research and support the development of standardized, evidence-based applications of IRT in bike fitting, while also highlighting its medical relevance in visualizing pressure zones and asymmetries that may help prevent overuse injuries and inform ergonomic design.

## 2. Methodology

### 2.1. Search Strategy and Study Selection

An initial exploratory search was conducted to identify relevant keywords and indexing terms. The formal search was performed using the databases Web of Science and PubMed, with the search terms ‘thermography’ AND ‘sport cycling’. This search yielded a total of 60 articles, with 33 articles retrieved from Web of Science and 27 from PubMed. Duplicate records were automatically identified and removed using EndNote’s duplicate detection tool, resulting in the exclusion of 13 duplicates. After deduplication, 47 articles remained for screening. Backward and forward citation screenings were initially considered; however, given the manageable number of results (60 initial articles), the database search was deemed comprehensive. We manually checked references of included papers to ensure no major study was missed, thereby mitigating the need for formal snowballing.

**Figure 2:**
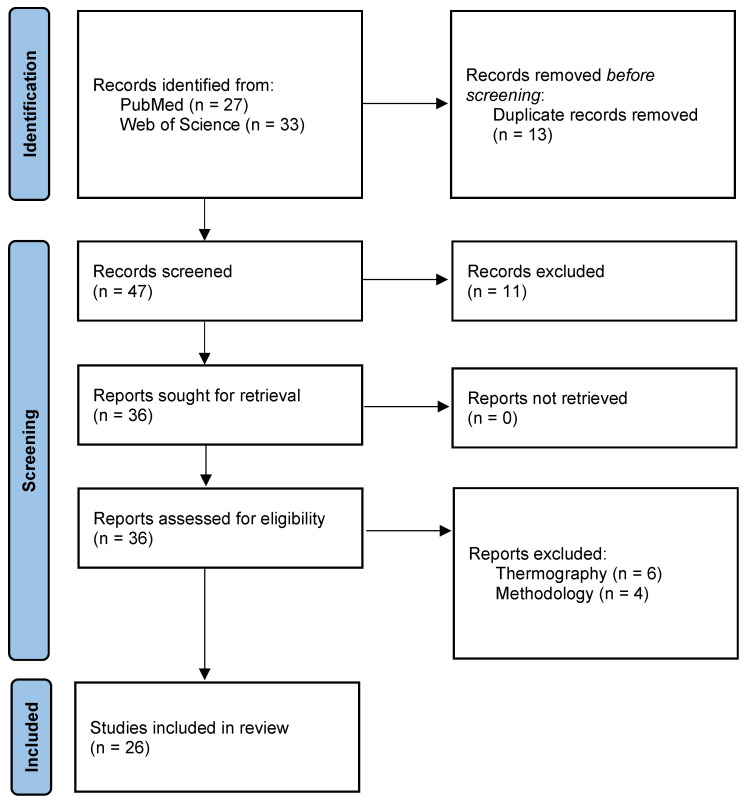
PRISMA flow diagram of the screening and selection process.

The titles and abstracts of the 47 articles were screened independently by two reviewers to assess their relevance. During this process, 11 articles were excluded as they did not align with the research focus. Consequently, 36 articles were selected for full-text assessment. All studies were successfully retrieved. After evaluating their eligibility, 26 studies met the inclusion criteria and were incorporated into the final analysis. All included studies were peer-reviewed English-language journal articles with available full texts, published between 2008 and 2024. The full screening and selection process are detailed in the PRISMA flow diagram, shown in Figure 2.

### 2.2. Inclusion Criteria

The eligibility assessment was based on both methodological quality and content relevance. Studies were included if they applied IRT in the context of sport cycling, specifically focusing on comfort, pressure points, or asymmetries. Articles were excluded if IRT was not used, cycling was not the primary activity studied, or essential methodological details were missing. Additionally, only studies published in English with full-text availability were considered.

The methodological quality and risk of bias of the included studies were assessed using the Newcastle-Ottawa Scale (NOS), which is a validated tool for evaluating non-randomized studies across the following three domains: selection of study groups, comparability of groups, and outcome assessment. To ensure sufficient methodological rigor, only studies with an NOS score of 7 or higher (out of 9) were included. Although all included studies met this threshold, selection bias remained a concern, as several studies focused on homogeneous populations (e.g., young, trained male athletes), limiting generalizability to broader groups such as recreational cyclists and female participants.

While different studies have employed various thermal camera brands (e.g., FLIR (FLIR Systems, Inc., Wilsonville, OR, USA), AVIO (Nippon Avionics Co., Ltd., Yokohama, Kanagawa, Japan), Fluke (Fluke Corporation, Everett, WA, USA) and AEG (AEG Power Tools, Winnenden, Baden-Württemberg, Germany)), as shown in Table 1, this did not affect key conclusions. Since asymmetries and pressure points were identified based on relative temperature differences rather than absolute values, variations in camera models did not influence the interpretation of findings.

### 2.3. Data Extraction

Data extraction was conducted independently by two reviewers using a standardized data extraction form. Extracted information includes the study title, authors, year, study objective, participant characteristics, camera type and positioning, measurement protocol, outcome measures, key findings, mentioned limitations, and conclusions. To ensure accuracy and consistency, the extracted data were cross-checked by a third reviewer. Discrepancies between the two primary reviewers were resolved through discussion, with the involvement of the third reviewer when consensus could not initially be reached.

To ensure consistent and reliable results, careful attention is paid to the selection of relevant body regions, known as regions of interest (ROIs), in the included studies. These regions, such as the knees, thighs, feet, and hands, are critical for evaluating thermal patterns and identifying pressure points. The ROI choices vary depending on the study’s objective, for example, studies focusing on detecting knee issues capture targeted thermal images of the knee joint [3], while studies investigating general pressure points often analyze multiple ROIs simultaneously [10,14,28]. In the context of bike fitting, obtaining precise thermal images of contact areas (saddle, pedals, handlebars) is crucial for identifying asymmetries and potential discomfort zones.

## 3. Results

### 3.1. Measurement Protocol

A consistent measurement protocol is crucial in thermographic studies to ensure reliable and reproducible results. Most reviewed studies have [10,13,14,20,29,30,31,32,33,34,35] adhered to standardized guidelines, which include restrictions on smoking, caffeine, and intense exercise before measurements. These precautions help minimize external influences on thermoregulation [36,37,38]. Additionally, acclimatization periods of 10 to 15 min were generally applied before measurement to establish a stable baseline temperature [31]. Interestingly, while some studies opted for shorter (5 min) or longer (30–40 min) acclimatization times [17,23], the consensus was that at least 10 min is essential for both male and female participants. Differences in warm-up protocols were also noted, with most cycling studies employing incremental power tests, starting at a low wattage and gradually increasing until exhaustion [10,16,17,18,29,32,33,34,35]. Thermal imaging was typically conducted before, immediately after, and at intervals during recovery to capture changes in skin temperature. None of the included studies specifically compared different bicycle frames or saddle designs using IRT. Thus, factors like the bike’s geometry or seat construction were held constant within each study’s context.

Table 2 provides an overview of the measurement protocols used in the included studies. For each study, the objective, corresponding ROIs, exercise protocols, and measurement procedures are presented. Additionally, information on the subjects, the thermal imaging camera used, and the camera-to-subject distance is included for completeness.

To ensure consistent and reliable results, careful attention was given to the selection of relevant body regions, known as ROIs, in the included studies. These regions, such as the knees, thighs, feet, and hands, are critical for evaluating thermal patterns and identifying pressure points. The ROI choices vary depending on the study’s objective; for example, studies focusing on detecting knee issues capture targeted thermal images of the knee joint [3], while studies investigating general pressure points often analyze multiple ROIs simultaneously [10,14,28]. In the context of bike fitting, obtaining precise thermal images of contact areas (saddle, pedals, handlebars) is crucial for identifying asymmetries and potential discomfort zones.

It is important to note that the included studies employed various infrared camera models (Table 2), including FLIR, AVIO, and Fluke, each differing in sensitivity and image resolution. FLIR cameras were the most frequently used (57.7% of studies), followed by AVIO and Fluke models (Table 1). Camera-to-subject distances ranged from 1 to 6 m, depending on the ROI under investigation. While variations in camera calibration and environmental conditions—such as ambient temperature and humidity—could influence absolute temperature readings, most studies focused on relative temperature differences, for instance in the identification of localized hot spots on the saddle. These relative measurements are generally robust against such variations, enhancing their reliability for comparative analysis. However, when interpreting absolute temperature values, these contextual factors should be carefully accounted for to ensure accurate and meaningful conclusions.

### 3.2. Effects on Skin Temperature

A recurring observation in the literature is the biphasic response of skin temperature during exercise, namely, an initial decrease due to vasoconstriction followed by a subsequent increase during recovery [34,35,39,40]. Vasoconstriction reduces peripheral blood flow to conserve heat and prioritize core organ function, resulting in lower temperatures in extremities during early exercise phases. Post-exercise, vasodilation facilitates heat dissipation, leading to increased skin temperature.

Differences in thermoregulation have been observed based on fitness levels, age, and gender. Trained participants exhibited more stable thermoregulation, higher skin temperatures during exercise, and faster recovery times compared to untrained individuals [22,35]. Similarly, elderly participants had lower resting temperatures and slower heat dissipation compared to younger individuals [41]. Gender differences were also significant, with women displaying greater temperature variability in certain regions post-exercise [13,31]. For example, Vieira et al. [13] noted that women’s skin temperature responses differed from men’s during recovery from resistance training, which may be attributed to differences in body fat and hormonal thermoregulation. A detailed overview of these key findings, including limitations and conclusions, is presented in Table 3.

Other factors influencing skin temperature include sweat production and body fat percentage. Sweating naturally cools the skin, reducing temperature readings, while body fat acts as an insulator, potentially affecting the accuracy of the results. This effect is particularly relevant in female participants, who typically have higher body fat percentages than men [34,42,43]. Although these physiological and environmental factors may influence absolute temperature values, their effect on relative temperature differences, which are commonly used in thermographic analyses, is considerably less pronounced. In practice, researchers often focus on temperature changes or differences (e.g., pre- vs. post-exercise, or left vs. right side) rather than absolute values, to account for these individual variations.

### 3.3. Symmetry Analysis

Symmetry in cycling posture is critical for both comfort and performance. Several studies have demonstrated that temperature asymmetries greater than 0.5 °C between corresponding body regions may indicate uneven weight distribution or improper posture [13,14,21]. Although symmetrical sports like rowing generally show fewer asymmetries [14,21], sports involving more unilateral movements can yield temperature differences of up to 1.7 °C [44]. A temperature difference of 1 °C is commonly considered a threshold for severe asymmetry, while smaller differences (0.3–0.4 °C) may still warrant further medical evaluation [19,45].

**Figure 3:**
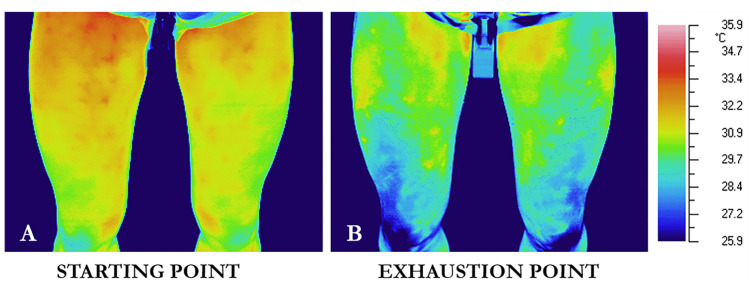
Bilateral thermal images pre- and post-exercise show symmetrical left–right temperature patterns, despite overall cooling with exertion [16].

Despite the promise of IRT for detecting such asymmetries, establishing a direct relationship between skin temperature asymmetry and muscle effort remains challenging in cycling. For instance, Trecroci et al. [16] found no significant correlation between bilateral crank torque differences and thigh skin temperature, suggesting that not all mechanical asymmetries manifest thermally. Nonetheless, bilateral thermal imaging before and after maximal exercise shows that highly trained cyclists typically exhibit symmetrical left–right patterns, even when absolute temperatures drop due to exertion (Figure 3). This suggests that trained individuals tend to cool evenly across both sides, with temperature asymmetries greater than 0.5 °C rarely observed.

**Figure 4:**
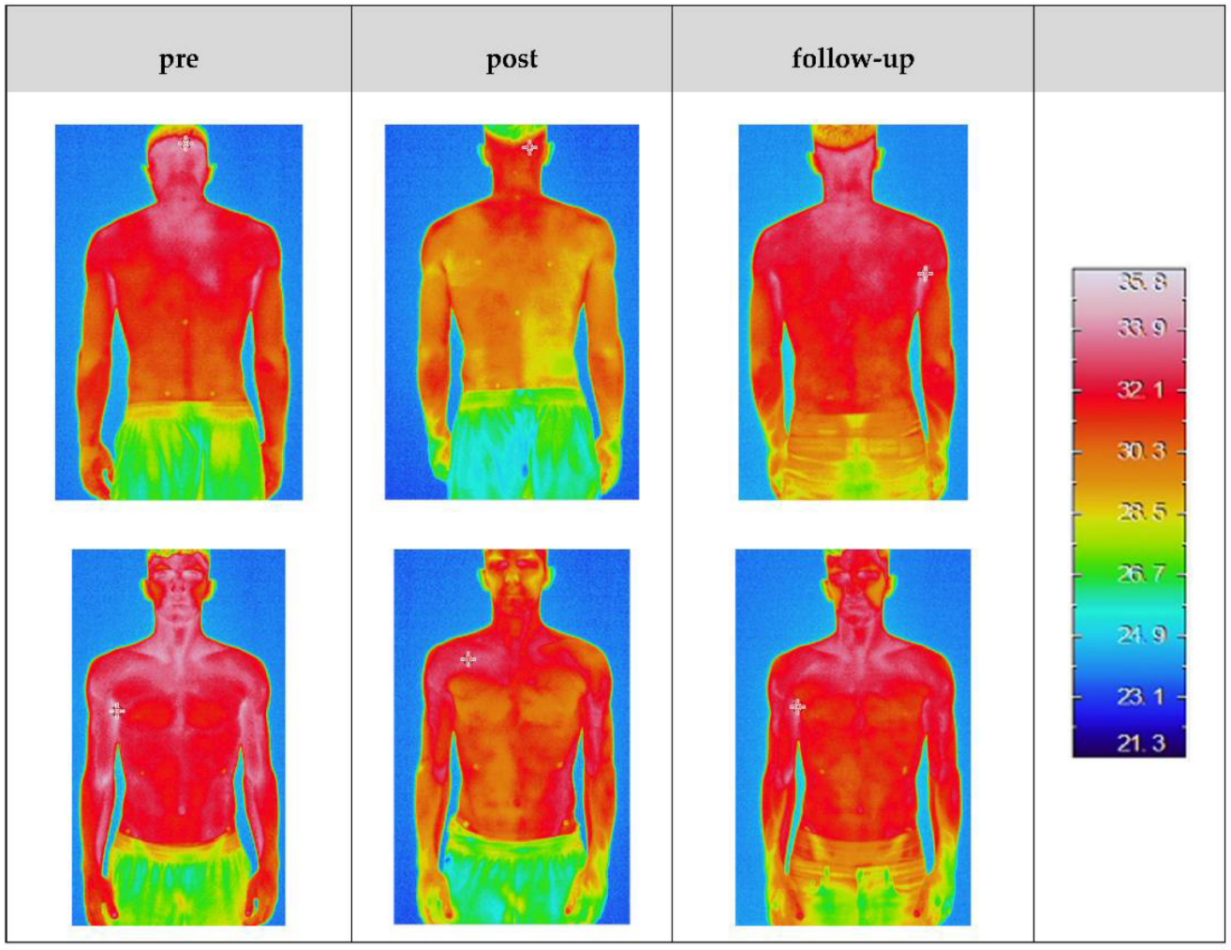
Thermal images of a subject’s back captured before (**left**), immediately after (**middle**), and 24 h after (**right**) unilateral fatigue of the trunk muscles. Post-exercise, a distinct temperature increase is visible on the treated (fatigued) side compared to the untreated side [12].

In contrast, thermal imaging may also reveal clear asymmetries under certain conditions. For instance, Dindorf et al. [12] reported that following unilateral back muscle fatigue, one side of the lower back exhibited noticeably higher temperatures than the other. Figure 4 presents thermal images of a single participant taken before exercise, immediately afterward, and after 24 h of recovery. The post-exercise image distinctly shows the right side of the lower back being warmer than the left side, clearly illustrating asymmetrical loading. This underscores that when one side of the body is subjected to increased stress, IRT can visually capture this asymmetry.

### 3.4. Injury and Pressure Point Detection

Several studies have focused on overuse injuries in cyclists—particularly knee joint strain and saddle-related soft-tissue discomfort—both of which are often linked to poor bike fitting [15,25,26,27]. Knee injuries are commonly associated with incorrect saddle height, with an optimal knee angle of 25° to 30° at the bottom dead center (BDC) recommended to reduce joint stress, although personal comfort and injury history should also be considered. The saddle region is another frequent source of discomfort due to sustained pressure, especially in female cyclists, for whom wider saddles have shown benefits [25,26,27]. Traditionally, pressure sensors have been used to assess peak saddle pressure and guide fitting adjustments, but IRT offers a complementary or alternative method by visualizing heat patterns that indicate pressure points and underlying stress [10,13,16,25,26,27,46]. IRT has been effective in detecting localized temperature increases at contact areas like the saddle and pedals, driven by mechanical compression, reactive hyperemia, and surface friction [13,16]. For instance, Bandeira et al. [15] detected muscle micro-injuries 24 h post-exercise using IRT, findings corroborated by elevated creatine kinase levels. However, not all IRT-indicated injuries were clinically confirmed, and external factors such as perspiration, ambient temperature, and individual thermoregulation can influence interpretation [42]. Unlike pressure sensors, which are mostly limited to saddle assessments, IRT can be applied more broadly to areas such as pedals and handlebars, where sensors are less commonly used [3,46,47].

**Table 2:** The objectives and corresponding measurement protocols of the included studies.

Study	Objective	Exercise Protocol	Measurement Procedure	ROI	Subjects	Camera and Position
Vieira et al. (2020) [13]	Analyze and compare skin temperature during resistance training in men and women; monitor thermal recovery	3 sets of 12 repetitions at 70–80%, 1 RM for lat pulldowns, leg presses, and biceps arm curl exercises	Interior and posterior body view at rest, 20 min and 24 h after training	Brachial biceps, quadriceps, and upper back	8 male and 8 female adults (24.56 ± 3.22 yrs) (healthy, no smoking, allergies, lotion, oils, creams before exercise)	FLIR T420 (320 × 240 pixels)
Szurko et al. (2022) [14]	Create a thermal map of the body surface in trained individuals	30 min adaptation, 3 min on rowing ergometer set at 90% capacity	Before and immediately after exercise, then 10 min after, 20 min after, 40 min after and 50 min after	12 ROIs: right wrist elbow flexor, left wrist flexor, biceps, triceps, pectoral muscles, anterior dentate muscles, right rectus muscle, trapezius muscles, gastrocnemius muscles, quadriceps, deltoid muscles, latissimus dorsi muscles	Six professional male adults (24–30 yrs) (no alcohol, stimulants, sauna, etc., before exercise)	FLIR T640 (640 × 480 pixels, distance of 3 m)
Bandeira et al. (2012) [15]	Evaluate the potential of thermography for diagnosing injuries caused by training	15 min adaptation, 2 groups: control group (low-intensity run 50–60% max heart rate), experimental group (80% heart rate squats, leg extension)	Image of quadriceps before training and another image taken 24 h after the session	Quadriceps	18 male football players (age: 15–17 yrs)	FLIR A-325 (320 × 240 pixels)
Trecroci et al. (2017) [16]	Examine the relationship between kinetic and thermal asymmetry during exercise	Adaptation to room temperature, 10 min warm-up at 100 W, increase 25 W every min until exhaustion	One image every 10 s, 1 before warm-up, 1 after warm-up, 1 at exhaustion point, and 2 after exercise (3 and 6 min after)	Independent pedal forces, peak crank torque, overall torque, skin temperature of quadriceps	10 male elite cyclists	AVIO TVS-700 (320 × 240 pixels, placed at a fixed height of 118 cm, background at constant temperature)
Quesada et al. (2016) [10]	Investigate how cycling postures, linked to knee angles, affect skin temperature, the reliability of thermal measurements in varied body regions	10 min adaptation; 3 tests of 45 min cycling at 50% peak power, each test in a different knee flexion 20, 30, 40° (when pedal crank at 180); one pre-test: 5 min warm-up at 50 W then 25 W added every min until exhaustion; main test: 3 min warm-up at 50 W, 45 min at 50%	Before, immediately after, and 10 min after the test	16 ROIs: chest, abdomen, upper back, lower back, vastus lateralis, rectus femoris, abductor, vastus medialis, biceps femoris, semitendinosus, knee, popliteal, tibialis anterior, gastrocnemius, ankle anterior, and Achilles	16 cyclists at club level (No smoking, no alcohol 12 h, no high intensity 24 h, and no lotions/creams 2 h before exercise)	FLIR E-60 (320 × 240 pixels, NETD < 0.05 °C)
Cholewka et al. (2015) [17]	Assess efficiency levels during endurance tests by comparing temperature parameters with data	30–40 min adaptation to room temperature; start at 50 W and every 3 min +30 W until exhaustion	Before, after, and every 3 min interval	Face, chest, arms, back, calf	12 healthy male cyclists	FLIR E-60 (320 × 240 pixels)
Ludwig et al. (2016) [18]	Assess temperature distribution on thighs during incremental exercise	10 min adaptation, 10 min warm-up at 100 W, then +25 W every min until exhaustion	Before the test, after warm-up, and after the incremental test (immediately, 3 min after, and 6 min after)	Thighs	7 male cyclists (no exercise 24 h before)	AVIO TVS-700 (320 × 240 pixels, set perpendicular to ROI)
Dindorf et al. (2022) [12]	Study the impact of asymmetric muscle fatigue on the skin temperature of abdominal/back muscles	Adaptation to room temperature; side bends on Roman chair in sets of 20 repetitions	Images were taken before, immediately after, and after 24 h	Side back and abdomen	41 subjects (22 male, 19 female; age: 22.63 ± 3.91 yrs; height: 173.36 ± 9.95 cm; weight: 71.89 ± 12.97 kg)	NEC AVIO TVS-200 (320 × 240 pixels)
Chudecka et al. (2015) [20]	Compare temperature changes of rowers and handball players after exercise	20 min warm-up, 2000 m rowing, and 90 min handball training	Two series: 20 min before training and immediately after training	Front and rear surfaces of the upper limbs (arm and forearm), chest, front and rear surfaces of thighs, the back	2 groups: 18 male scullers, average age of 20.77 yrs, and handball players (no specific information)	FLIR ThermaCAM SC500 (256 × 256 pixels, with a distance of 3 m; conditions: 25 °C and 60% humidity)
Formenti et al. (2012) [22]	Examine differences in skin temperature changes after exercise between trained and untrained individuals	15 min adaptation, standing heel raises for 2 min	Before exercise (1 min), during exercise (2 min), and after exercise (7 min)	Lower limbs (calves, Achilles tendon)	7 sedentary female subjects (no alcohol and caffeine 4 h before exercise)	AVIO TVS-700 (320 × 240 pixels)
Bayrak et al. (2024) [23]	Determine the level of participation in the training of an athlete with sartorius muscle injury using IRT	5 min adaptation, then cycling at 30–40% intensity because he was in pain	Daily before and after exercise	Sartorius muscle	1 professional football player (23 yrs, 185 cm, 81 kg)	Not specified
Straburnzyńska-Lupa et al. (2022) [25]	Assess elite rowers’ skin temperature; examine the link between resting skin temperature and muscle peak torque	15 min adaptation, rowing (not specified)	Before, immediately after, and 15 min after exercise	Back and front body A-pose	10 professional male sweep rowers (no caffeine/coffee, no alcohol, no lotion/oil before exercise)	FLIR SC640 (640 × 380 pixels, height of 1 m, distance of 6 m)
Duc et al. (2015) [29]	Analyze the relationship between body/muscle efficiency and skin thermograms during graded cycling	Start at 100 W and every 4 min +40 W until exhaustion	Taken throughout exercise from 5 min before to 5 min after	Right calf, left quadriceps	7 young, healthy male competitive cyclists (no smoking, no alcohol, no coffee/caffeine 3 h, no intense exercise 24 h, and no cream, lotion or gel 2 h before exercise)	FLIR SC1000 (256 × 256 pixels, height of 0.5 m and distance of 1.5 m at the right side of the vehicle)
Ludwig et al. (2013) [30]	Compare methods for analyzing thermographic images	15 min adaptation, staying in an anti-gravitational static position	3 images of the calves, 1 every 20 s	Calves	33 subjects (male/female) (healthy, no drugs, alcohol, caffeine for 4 h before exercise, no body hair)	AVIO TVS-700 (320 × 240 pixels)
Marins et al. (2014) [31]	Determine acclimation time needed to achieve thermal balance in young individuals at rest	Standing for 20 min, no sitting, crossing arms, or scratching	Every 2 min, 4 images (in total 20 min)	Hands, forearms, arms, thighs, legs, chest, and abdomen	44 subjects: 18 men (22.3 ± 3.1 yrs) and 26 women (21.7 ± 2.5 yrs) (no pain, no medication 2 weeks, no smoking, no cream/lotion 6 h, no exercise 24 h before exercise)	Fluke ATIR-25 (160 × 120 pixels)
Arfaoui et al. (2014) [32]	Analyze the relationship between gastrocnemius skin temperature and heart rate during incremental cycling	5 min sitting, cycling at 100 W for 10 min, then +50 W every 3 min at up to 200 W, then 250 W for 2 min, 30 min was needed to balance the body’s temperature with the environment before resuming	Not specified	Calves	11 male cyclists (15 ± 11 yrs experience) (no smoking, alcohol, coffee/caffeine for 6 h before exercise)	FLIR SC1000 (256 × 256 pixels)
Quesada et al. (2016) [33]	Compare skin temperature differences between cyclists and non-cyclists, relationship with performance factors	3 min warm-up at 105 W, then 35 W increase per 3 min until exhaustion; 10 min adaptation before the test; after 10 min adaptation, immediately after the test and 10 min after the test	Not specified	Vastus lateralis, rectus femoris, biceps femoris, and gastrocnemius medialis	11 cyclists and 11 non-cyclists (no alcohol, coffee/caffeine, smoking 12 h, exercise, no sunbath, no creams, lotions, oils before exercise)	FLIR T420 (320 × 240 pixels, perpendicular to ROI, distance of 1 m, no electronic devices in 5 m radius)
Ferreira et al. (2008) [41]	Evaluate thermographic changes linked to localized exercise in young and elderly participants	10 min adaptation, warm-up exercise for right lower limb: isotonic exercises of knee extension/flexion with 1 kg weight resistance above the ankle, 3 min full range of motion	Pre-exercise, immediately post-exercise, and during the 10-min period post-exercise (every 2 min 5 times)	Posterior thigh’s skin temperature	14 elderly (67 ± 5 yrs) and 15 young (23 ± 2 yrs) healthy subjects (no food 2 h, no alcohol or exercise 24 h, no lotion 2 h before exercise)	Not specified (height of 36 cm, distance of 2.34 m)
Vardasca et al. (2016) [48]	Assess thermal symmetry in the extremities of participants	Room adaptation of 15 min	Not specified	Total body anterior, total body dorsal, right arm, left arm, both hands, thighs, lower legs, plantar feet, and dorsal feet views	39 males (26.9 ± 10.2 yrs) (no smoking, no heavy meal, no alcohol 2 h, no exercise, no oils/lotion before exercise)	FLIR A40 (320 × 240 pixels)
Mu noz et al. (2024) [44]	Evaluate skin temperature asymmetries in padel players; associations with fatigue, pain, and experience	Training of 3 ± 1 h with a common training pattern	Before exercises, immediately after, and 10 min after	Upper limbs: anterior shoulder, anterior arm, anterior forearm, abdominal, posterior shoulder, posterior arm, posterior forearm, and lower back; lower limbs: anterior thigh, anterior knee, anterior leg, posterior thigh, posterior knee, and posterior leg	10 professional padel players (9 males, 1 female) (no UV treatment 12 h, no medication, no body cream before exercise)	FLIR E60 (320 × 240 pixels, perpendicular to ROI, distance of 2 m)
Salamunes et al. (2017) [42]	Investigate the relationship between body fat percentage and skin temperature	15 min adaptation, no exercise, participants were grouped based on %BF	Not specified	Anterior and posterior views, on both right and left sides: arms, forearms, thighs, shanks, palms, abdomen, and flanks	123 Women (18–35 yrs, BMI: 18.5–29.99 kg/m^2^) (not pregnant, no fever for 15 days, no alcohol, caffeine/coffee, lotions, deodorants, no smoking before exercise)	Fluke Ti400 (320 × 240 pixels, orthostatic position, distance of 2.5 m)
Zontak et al. (1997) [34]	Define skin temperature responses to exercise using thermography	15 min adaptation; method 1: cycling with a load increase of 50 W every 3 min until exhaustion; method 2: rest and steady state (640 s at rest, 1280 s under load)	Not specified	Method 1: hand skin temperature under conditions of rest, load, and recovery; method 2: dynamics of hand surface temperature under rest followed by constant load	10 subjects (age: 25.8 ± 0.7 yrs, height: 175.3 ± 1.8 cm, weight: 70.1 ± 2.5 kg)	Not specified
Merla et al. (2005) [35]	Examine differences in skin thermoregulation during graded exercise between trained and untrained individuals	High-resolution thermal video: thighs recorded at rest for 1 min, during exercise and along the recovery phase	High-resolution thermal video: thighs recorded at rest for 1 min, during exercise and along the recovery phase	Total body	18 young healthy men (19–26 yrs), 10 professional footballers and 8 sedentary subjects (non-smokers, no drugs)	AEG Aim 256 PtSi (256 × 256 pixels)
Cuevas et al. (2014) [28]	Use IRT to monitor changes in muscle and joint temperature after exercises, understanding effects on thermoregulation and metabolism	Strength training session protocol: 5 min warm-up bicycle + stretch, 4 sets of 10 reps at 70%, 90 s rest, bench, leg press, leg extensions, crossed pulleys; also 5 min warm-up, 45 min treadmill 60–75%. 15 min adaptation, no exercise 24 h, comfortable clothing	10 series of thermograms before, immediately after, and one hour after exercise	Pectoral, dorsal, deltoids, biceps brachii, triceps brachii, quadriceps, and hamstring muscles, the elbow and knee joints bilaterally, abdominal	15 physically active students	FLIR 335 (320 × 240 pixels)
Tanda et al. (2015) [39]	Evaluate skin temperature responses to controlled running exercise	10 min adaptation, running exercise on a treadmill with graded or constant load	During different phases of exercise, including warm-up, running at a constant velocity, and resting periods	Anterior thigh, abdomen, posterior calf, etc.	7 healthy and active subjects (6 males and 1 female)	FLIR T335 (320 × 240 pixels)
Hadzic et al. (2015) [45]	Study the connection between skin temperature changes and muscle fatigue, determine the usefulness of thermography for monitoring fatigue	15 min adaptation, 6 min warm-up at 100 W, 10 s stretch, then 7.5 min at 120°/s on a dynamometer	Not specified	Quadriceps	1 male (23 yrs, 12 yrs training history, 178.5 cm, 68 kg)	FLIR T425 (320 × 240 pixels)

**Table 3:** The key findings, limitations, and conclusions of the included studies.

Study	Key Findings	Limitations	Conclusions
Vieira et al. (2020) [13]	Women had significantly lower skin temperature than men, with no significant differences between sexes at both 20 min and 24 h after exercise; asymmetry is noted when the temperature difference exceeded 0.5 °C	The type of strength training performed may induce thermal changes 24 h after exercise compared to baseline values in the ROIs, these areas would thus require a longer recovery time	Women’s resting skin temperature was lower than men’s across all ROIs; strength training did not alter baseline skin temperature at 20 min post-exercise in the analyzed regions; 24 h post-training, men showed increases compared to base values in upper back skin temperature, women in quadriceps
Szurko et al. (2022) [14]	An initial decrease in skin temperature due to vasoconstriction; an increase in skin temperature in specific regions when blood flow drifts from internal tissues to the skin surface	Not specified	Thermography can assess muscle symmetry; temperature differences did not exceed 0.5 °C, meaning no significant asymmetry; thermal imaging is a non-contact, safe, and innovative method for verifying muscle symmetry
Bandeira et al. (2012) [15]	The experimental group experienced some micro-injuries, triggering an inflammatory process, notable muscular temperature gradient was observed 24 h after exercise	Not specified	Thermography, alongside creatine kinase, can non-invasively detect and localize muscle injuries post-training; fat layer thickness affects accuracy
Trecroci et al. (2017) [16]	Significant asymmetry in peak crank torques, the right limb is stronger and colder, no correlation between asymmetry and skin temperature differences, fewer asymmetries with highly trained cyclists, sweat production can reduce skin temperature	The experimental setting is far from regular training or competition environments, with no pedal power measurements, only analyzing the quadriceps	Bilateral differences in kinetic variables did not correspond to skin temperature differences during a maximal incremental cycling test, challenging to link skin temperature with muscle effort, future studies should include additional cameras to analyze other areas of the lower limbs
Quesada et al. (2016) [10]	Changing saddle height did not lead to changes in skin temperature at the regions of interest, different cycling postures from saddle height variations had no impact on thermal measurements	Sweat on the skin could influence the IRT data as it may work as a filter	Changes in knee flexion angles did not alter skin temperature at the ROIs, application of IRT for analyzing saddle height effects does not seem suitable
Cholewka et al. (2015) [17]	A strong negative correlation was found between average power output and average body surface temperature from thermal parameters	Not specified	Thermal imaging can be useful for evaluating athletic efficiency, it may also aid in training assessments
Ludwig et al. (2016) [18]	Heat-spot patterns on thighs linked to effort type rather than exercise type	Controlled environment conditions	Decrease in temperature post-exercise, rapid recovery immediately after; hot-spotted thermal pattern on the skin surface
Dindorf et al. (2022) [12]	Temperature changes were visible post-test, decreasing after 24 h; back muscles showed asymmetric skin temperature differences post-test, with the treated side slightly warmer	Body fat influences thermographic reactions [14], laboratory environmental conditions	Thermography is effective for detecting differences between treatment and non-treatment areas, suggesting potential in evaluating the effectiveness of treatment for asymmetric muscle fatigue; thermography can bring value in diagnosing muscular imbalances
Chudecka et al. (2015) [20]	Scullers: mean skin temperature was lower post-exercise than pre-exercise, symmetrical muscle activity; handball players: significant differences in skin temperatures between symmetric and asymmetric muscle areas	Small sample size, lack of detailed information on the handball players	Thermal imaging could be a valuable tool for assessing technical preparations in sports requiring symmetrical muscle use, like rowing; symmetry is crucial for optimal performance in such sports
Formenti et al. (2012) [22]	Female athlete’s skin temperature increased more during steady-load exercise compared to sedentary female controls	Heat dissipation is influenced by variables like body hair and fat distribution	Physical training influences the rate of skin temperature increases during localized exercise in female subjects; IRT has the potential to track skin temperature changes during exercise and is helpful in studying skin temperature changes and their physiopathology
Bayrak et al. (2024) [23]	Temperature increased significantly after 10 min of cycling at 30–40% intensity	Not specified	Thermography is useful for detecting muscle injuries and can be employed in injury prevention strategies
Straburnzyńska-Lupa et al. (2022) [25]	No significant temperature asymmetries were found	Not specified	No significant temperature asymmetries were found; thermal imaging can effectively track changes in skin temperature and symmetry before and after exercise
Duc et al. (2015) [29]	Strong correlations were observed between changes in heart rate, oxygen uptake, and skin temperature in the vastus lateralis and gastrocnemius medialis muscles	Efficiency is calculated at the same workload but not at identical pedaling cadence, the time of power output steps to measure data is much lower than recommended	Significant inverse correlation between skin temperature changes in vastus lateralis and cycling efficiency, cyclists with greater skin temperature decrease showed better thermoregulation during exercise
Ludwig et al. (2013) [30]	Comparison of three IRT methods (Troi, Tmax, Ttot) for skin temperature analysis; Troi and Ttot yield similar values, while Tmax detects higher temperatures and asymmetry more effectively	Small sample size, limited to calf muscles, only static images analyzed	All three methods effectively capture skin temperature trends, Tmax is the most suitable for detecting asymmetry and dynamic conditions
Marins et al. (2014) [31]	Women exhibited significantly higher temperature variations than men; men: significant temperature variation in the abdomen; women: variations in the anterior abdomen, thighs, and posterior parts of the hands and forearms	Not specified	The time required to reach skin temperature balance in young men and women is variable, 10 min of rest for whole-body thermal analysis is recommended for both sexes
Arfaoui et al. (2014) [32]	No asymmetries were found	Not specified	A significant correlation was found between heart rate and skin temperature evolution during graded exercise, no asymmetries were detected
Quesada et al. (2016) [33]	Cyclists had lower body fat percentage, higher peak power output, higher oxygen consumption, higher heat production, and higher skin temperatures in knee extensors compared to non-cyclists; skin temperature was negatively correlated with body fat and positively correlated with peak power output and heat production	Sweat on the skin could have influenced the thermal data due to its effect as a filter for infrared radiation [49]	Cyclists had higher skin temperatures than non-cyclists during and after an incremental cycling test, heat production is a key variable to consider when interpreting skin temperature results, skin temperature dynamics are influenced by body composition and cycling performance, and maintaining low body fat is recommended for improved heat dissipation
Ferreira et al. (2008) [41]	No temperature differences were observed pre-exercise, younger individuals had higher baseline limb temperatures than elderly participants, post-exercise: the temperature in the exercised limb was greater	No similar health conditions between subjects	IRT is effective in detecting thermal responses during low-exercise conditions, young subjects had higher resting temperatures than the elderly, elderly showed slower heat dissipation post-exercise despite similar heat production
Vardasca et al. (2016) [48]	The greatest difference in standard variations between the contralateral areas was found to be 0.12 °C (forearm, anterior view); the asymmetry threshold is at 0.5 °C	Not specified	Regional views provided better thermal symmetry than full-body views, particularly for hands and feet, with excellent thermal symmetry in healthy individuals, with maximum differences of 0.4 °C
Mu noz et al. (2024) [44]	No asymmetries before exercise; post-exercise differences found in anterior and posterior forearms, shoulders, and arms; 10 min post-exercise, differences in anterior and posterior arms and forearms; strong correlation found between thermal asymmetries in the knee and racket weight (moderate correlation between the posterior thigh and age)	Small sample size, only one female; training was the same for all players, so load cannot be objectively determined	Asymmetries were observed in upper limbs post-training, increasing during recovery (10 min post-exercise); skin temperature dominance was not related to changes in fatigue, pain, or years of experience, but was associated with racket mass in the anterior knee and age in the posterior thigh; thermography can be useful for observing asymmetries
Salamunes et al. (2017) [42]	Higher body fat percentage correlated with lower skin temperatures in posterior thighs, shanks, and arms; body fat percentage positively correlated with palm temperatures and body circumferences	The lack of control of subjects’ menstrual cycle phases may alter thermal results [37]	Skin temperature distribution is influenced by body fat percentage, future studies should consider body fat’s impact on heat dissipation
Zontak et al. (1997) [34]	Method 1: hand temperature decreased over time; method 2: steady-state temperature at rest reached after 500 s; higher initial fingertip temperatures correlated with greater rates of decrease	Not specified	Rate of temperature decrease was influenced by the initial hand temperature; graded load exercises caused continuous cooling of fingers; steady-state exercises caused a similar decrease, followed by rewarming post-exercise
Merla et al. (2005) [35]	Untrained subjects showed lower minimal skin temperature after exercise compared to trained subjects, trained subjects had better blood circulation, skin temperature decreased in both trained and untrained groups during exercise due to continuous vasoconstriction as exercise intensity increased	Not specified	Trained individuals showed better vasoconstriction and heat dissipation during exercise; trained group was more efficient at transferring heat from muscles to skin
Cuevas et al. (2014) [28]	Recovery times varied by muscle group, exercise type, and intensity	Only 15 participants, a controlled environment, and limited exercises	IRT could offer valuable insights into temperature changes as a reaction to exercise; it is a safe, effective tool for monitoring activity and recovery patterns
Tanda et al. (2015) [39]	Early decrease in skin temperature during graded load exercise due to vasoconstriction, relative minimum during constant load, gradual increase during recovery	A limited number of subjects, controlled laboratory conditions	Human thermoregulation during running is influenced by dynamic energy balance and environmental and physical work conditions; IRT is effective for real-time, non-invasive monitoring of skin temperature; skin temperature decreases initially during exercise due to vasoconstriction
Hadzic et al. (2015) [45]	Negative correlation between quadriceps skin temperature increase and power decrease	Not specified	Possible relationship between skin temperature changes and muscle fatigue, monitoring skin temperature could provide an easier tool for tracking muscle fatigue

## 4. Discussion

### 4.1. Thermography vs. Traditional Bike-Fitting Methods

Traditional bike fitting primarily relies on biomechanical assessments, focusing on parameters such as joint angles, limb alignment, and body positioning, often using tools like video analysis or 3D motion capture systems. While these methods are well-established and effective for evaluating movement patterns, they offer limited insight into the physiological responses of the rider—such as pressure distribution, asymmetries, and localized discomfort—that can significantly influence both comfort and performance. These aspects, although critical, are not always apparent through kinematic data alone.

IRT presents a valuable complementary approach by offering non-invasive, real-time insights into the thermal responses of the body during and after cycling. By visualizing heat patterns, IRT can highlight areas of excessive pressure, thermal asymmetry, or regions susceptible to discomfort or overuse injuries—patterns that often remain undetected in conventional biomechanical analyses [10,13,14,16,17,20,35]. Most reviewed studies confirmed IRT’s ability to detect relative temperature changes linked to fatigue or discomfort, with consistent findings such as post-exercise hyperemia and asymmetries exceeding 1 °C frequently aligning with known mechanical issues (e.g., cleat misalignment) [13,16,20]. However, some limitations must be considered: temperature readings can be affected by factors such as body fat insulation and sweat-induced cooling, necessitating standardized environmental controls and protocols to ensure measurement consistency [20,34]. Moreover, IRT does not provide direct biomechanical data, such as joint kinematics or muscle activation patterns. Its utility, therefore, lies not in replacing traditional methods but in enhancing them. For example, while IRT can pinpoint a hot spot on a saddle, a traditional pressure sensor can quantify the pressure at that spot; it remains unclear whether IRT’s qualitative insight leads to better adjustments than the bike fitter’s usual process. No direct head-to-head studies between IRT-guided fitting and conventional fitting were found. Therefore, we cannot claim that IRT is more effective than traditional methods, only that it provides additional information. The findings underscore the importance of integrating IRT with biomechanical tools to gain a more comprehensive understanding of cyclist fit and function. Notably, not all biomechanical deviations manifest thermally—Trecroci et al. [16], for instance, found no thermal asymmetry associated with crank torque imbalance—highlighting that thermographic findings should always be interpreted in conjunction with mechanical data for accurate and meaningful conclusions.

### 4.2. Practical Applications of Thermography in Bike Fitting

While IRT shows promise for applications in bike fitting, its potential must be evaluated with caution. IRT provides real-time visualizations of pressure-related heat patterns, asymmetries, and possible areas of discomfort, which can support immediate adjustments aimed at enhancing rider comfort and performance. For instance, thermal hotspots on the saddle or pedals may suggest localized overloading that, if unaddressed, could lead to discomfort or injury over time [10,13,16,25,26,27,46]. However, such thermal indicators are inherently qualitative and indirect. Without concurrent biomechanical data—such as joint kinematics or quantified pressure—thermographic findings alone may lack the specificity required for targeted and effective interventions.

In high-level or professional settings, IRT may contribute to more individualized fitting by accounting for a rider’s unique physiological responses. Nonetheless, its role should be viewed as complementary rather than standalone. Not all biomechanical dysfunctions produce detectable thermal asymmetries, and some thermographic patterns may be influenced by non-mechanical factors, including body composition or environmental conditions. This underscores the necessity of interpreting IRT results alongside objective biomechanical assessments.

Although several challenges persist, including the absence of standardized protocols and the requirement for expertise in data interpretation, the relatively low cost and non-invasive nature of thermographic equipment make IRT an accessible and scalable complement to current bike-fitting technologies. Nevertheless, in the absence of direct comparative studies, it remains unclear whether IRT-guided fitting leads to superior outcomes compared to conventional approaches. Therefore, IRT should be integrated carefully into a broader, evidence-based assessment strategy, rather than viewed as a substitute for established biomechanical methods.

### 4.3. Limitations

Because camera settings and measurement protocols varied between studies, direct comparison of absolute quantitative temperature values should be approached with caution. Differences in thermal sensitivity, calibration, or environmental conditions (e.g., room temperature, humidity) may have influenced the magnitude of the reported temperature readings. However, such factors are less likely to affect the interpretation of relative temperature differences, which were the primary focus in most studies. The lack of standardized control over physiological and environmental variables—such as hydration status, sweat rate, and ambient temperature—represents a limitation. While these factors primarily affect absolute values, they may also introduce subtle variability in relative temperature patterns, especially in cross-study comparisons. This potential influence should be acknowledged, as it may have impacted the conclusions of this review.

A meta-analysis was not conducted due to substantial heterogeneity in study designs, outcome measures, and reporting styles. Many studies presented qualitative trends rather than comparable quantitative metrics, which precluded the calculation of a meaningful pooled effect size. Consequently, the conclusions of this review are based on recurring patterns observed across studies, rather than on aggregated statistical evidence. This approach allows for the identification of general tendencies but does not yield definitive conclusions. For example, while several studies reported temperature changes in the range of 0.5 to 1.0 °C associated with fatigue, the absence of uniformly controlled comparison groups limits the statistical generalizability of such findings. Overall, this systematic review identifies consistent trends and proposes hypotheses—such as the potential of IRT to detect asymmetry-related issues—but does not provide definitive quantitative proof. These preliminary findings should be validated through well-controlled, hypothesis-driven studies.

This review is constrained by the limited scope of existing studies. Notably, many included experiments involved homogeneous groups of young, trained male cyclists, raising concerns about selection bias. Thermal responses and fitting issues in female or recreational cyclists might differ—for example, women may experience different saddle pressure distributions—yet such groups were underrepresented. Consequently, the findings may not generalize to all cyclist populations.

Study quality was assessed using the NOS, which provided a standardized framework to ensure baseline methodological rigor. Nonetheless, the NOS has recognized limitations; while it emphasizes aspects such as selection and comparability, it may overlook certain sources of bias, including lack of blinding or insufficient detail in intervention reporting. As a result, potential biases—such as measurement bias or unreported confounding variables—may have been present in some included studies without being adequately captured by the NOS scoring system.

### 4.4. Recommendations for Future Research

The reviewed studies demonstrate the promise of IRT in enhancing bike-fitting practices, yet several gaps remain. A critical next step is to determine whether the additional information provided by IRT actually leads to better outcomes. While IRT can visualize thermal patterns indicative of pressure or asymmetry, it remains unclear whether these insights translate into more effective adjustments than those achieved through conventional fitting approaches. Therefore, future research should explicitly compare the outcomes of fittings conducted with and without IRT guidance—assessing variables such as rider comfort, power output, and injury incidence—to establish its practical value in bike fitting. Such studies would help move the field beyond proof-of-concept and clarify IRT’s potential role in evidence-based practice.

In parallel, further efforts should aim at developing standardized measurement protocols, including detailed guidelines for camera positioning, acclimatization periods, and environmental conditions. This would enhance consistency across settings and improve comparability between studies. Moreover, it is essential to investigate the correlation between thermographic findings and subjective comfort ratings. Establishing a clear link between observed thermal asymmetries and perceived discomfort would support more targeted interventions and help validate IRT as a decision-making tool.

This review focused primarily on rider-related thermal patterns; however, the influence of equipment characteristics—such as saddle design or material—on these patterns remains underexplored. Understanding how such factors modulate thermal responses could offer new perspectives on bike-equipment interactions and their implications for fit.

Another critical area for future research involves the inclusion of a balanced representation of male and female participants. Given the physiological and anatomical differences between genders, particularly in terms of pressure distribution and comfort needs, ensuring gender diversity in studies will enhance the generalizability of findings and improve the applicability of IRT in bike fitting.

Moreover, dynamic assessments using thermal videography during real-time cycling could provide valuable insights into how thermal patterns evolve under varying conditions, such as changes in speed, terrain, or cycling posture. This approach may reveal previously undetectable issues related to biomechanical imbalances or pressure points during dynamic movement, further enhancing the precision of bike fitting.

Finally, longitudinal studies are necessary to evaluate the long-term impact of IRT-informed bike fitting on cycling performance, injury prevention, and overall comfort. By including a diverse range of participants, from recreational cyclists to elite athletes, future research can validate the effectiveness of IRT in both professional and everyday cycling contexts. Addressing these research areas will not only bridge the gap between laboratory findings and real-world applications but also contribute to more effective and data-driven bike-fitting solutions.

## 5. Conclusions

This paper has highlighted the potential of IRT as a valuable addition to current bike-fitting practices. While traditional methods focus primarily on biomechanical alignment, IRT offers a non-invasive means to capture physiological responses such as pressure distribution and asymmetry, factors closely linked to comfort and injury risk. By providing real-time, objective insights that go beyond what kinematic analysis can reveal, IRT can enhance the precision and effectiveness of rider assessments. Its integration into bike fitting, however, depends on the development of standardized protocols for measurement, interpretation, and application. With such frameworks in place, IRT could play a key role in advancing towards a more holistic, data-driven approach to cycling ergonomics.

## Figures and Tables

**Table 1:** The distribution of thermal camera types across the included studies.

Thermal Camera Type	Percentage of the Included Studies (%)
FLIR	57.7
AVIO	19.3
Fluke	7.7
AEG	3.8
Not mentioned	11.5

## Data Availability

No new data were created.
